# Aging into Perceptual Control: A Dynamic Causal Modeling for fMRI Study of Bistable Perception

**DOI:** 10.3389/fnhum.2016.00141

**Published:** 2016-03-31

**Authors:** Ehsan Dowlati, Sarah E. Adams, Alexandra B. Stiles, Rosalyn J. Moran

**Affiliations:** ^1^Virginia Tech Carilion School of MedicineRoanoke, VA, USA; ^2^Virginia Tech Carilion Research InstituteRoanoke, VA, USA; ^3^Virginia Commonwealth University School of MedicineRichmond, VA, USA; ^4^Bradley Department of Electrical and Computer Engineering, Virginia TechBlacksburg, VA, USA

**Keywords:** visual illusion, visual processing, aging, dynamic causal modeling, fMRI

## Abstract

Aging is accompanied by stereotyped changes in functional brain activations, for example a cortical shift in activity patterns from posterior to anterior regions is one hallmark revealed by functional magnetic resonance imaging (fMRI) of aging cognition. Whether these neuronal effects of aging could potentially contribute to an amelioration of or resistance to the cognitive symptoms associated with psychopathology remains to be explored. We used a visual illusion paradigm to address whether aging affects the cortical control of perceptual beliefs and biases. Our aim was to understand the effective connectivity associated with volitional control of ambiguous visual stimuli and to test whether greater top-down control of early visual networks emerged with advancing age. Using a bias training paradigm for ambiguous images we found that older participants (*n* = 16) resisted experimenter-induced visual bias compared to a younger cohort (*n* = 14) and that this resistance was associated with greater activity in prefrontal and temporal cortices. By applying Dynamic Causal Models for fMRI we uncovered a selective recruitment of top-down connections from the middle temporal to Lingual gyrus (LIN) by the older cohort during the perceptual switch decision following bias training. In contrast, our younger cohort did not exhibit any consistent connectivity effects but instead showed a loss of driving inputs to orbitofrontal sources following training. These findings suggest that perceptual beliefs are more readily controlled by top-down strategies in older adults and introduce age-dependent neural mechanisms that may be important for understanding aberrant belief states associated with psychopathology.

## Introduction

Several studies have demonstrated that later patient age-at-onset is a predictor of greater remission rates and better outcome prognosis in psychopathologies including schizophrenia (Häfner et al., 1998; Ho et al., 2000; Jeste et al., 2003), first-episode psychosis (Malla et al., 2006) and bipolar disorder (Carlson et al., 2002; Carter et al., 2003), independent of other contributing factors such as illness duration. Age also influences the relative symptom spectrum in these psychopathologies (Gur et al., 1996; Topor et al., 2013). In schizophrenia, for example the trajectories of positive, negative and thought-disorder symptom dimensions have been shown to display differential age effects, with advancing age associated with decreases in positive symptoms including hallucinations, delusions and bizarre behavior (Schultz et al., 1997). However, the putative neural mechanisms underlying adaptive effects of aging have been relatively unexplored in the neuroimaging and neuropsychiatric literature.

For this special issue on psychopathology, we aimed to address the basic mechanisms of brain networks that underlie age-dependent changes in constructive perception. A method of examining conscious perception is to take advantage of the visual system by instigating bistable perception. This allows us to study the underlying neural networks related to perception formation rather than stimulus-driven visual processing. Illusory visual paradigms have proved useful in probing the neural mechanisms associated with impaired perceptual inference and aberrant beliefs in psychosis and schizophrenia (Foxe et al., 2005; Dima et al., 2009, 2010; Notredame et al., 2014). Ambiguous visual stimuli such as the Necker’s cube, Rubin’s face-vase, or Boring’s Old-Young lady, where images have two distinct interpretations (Leopold and Logothetis, 1999), in particular lend themselves to the study of volitional inference and subjective perception (Sundareswara and Schrater, 2008; Wang et al., 2013). Moreover, these paradigms are often designed to illicit activations across distributed cortical networks or hierarchies. Earlier theories of switching perceptions focused on neuronal adaptation as a key mediator (Blake, 1989) however these have been superseded by connectivity analyses which demonstrate that bottom-up and top-down connections to early visual cortices (Cardin et al., 2011; Wang et al., 2013) and endogenous neuronal oscillations (Kloosterman et al., 2015) also contribute to the bistability of a percept. Bayesian decision theory, used to construct models of perception (Kersten and Schrater, 2002) support the role of networked cortical communication. In these accounts, reverses in perception between competing alternatives are posed as an active process that involves multiple regions of the brain seeking to understand the stimulus, where one particular perception emerges as the result of bottom-up and top-down interplay that suppresses one interpretation in favor of the other (Dayan, 1998). Modeling accounts have also demonstrated a potential impact from noisy neuronal firing as a possible bottom-up influence in perceptual switches (Shapiro et al., 2009). These computational accounts appeal to priors on what might be perceived—on our visual beliefs (Cardin et al., 2011).

In terms of the prior beliefs that encourage perceptual switching and image stability, opposing behaviors have been observed which support both bottom-up and top-down neuronal mediators. Some studies reveal that the most prevalent percept in the recent past is the one that is most likely favored when the ambiguous image is shown (Leopold et al., 2002), suggesting that implicit perceptual memory may affect perception of ambiguous figures (de Jong et al., 2014). Other studies have shown that prolonged viewing of an ambiguous stimuli leads to preference of the novel perception vs. past perceptual experience (de Jong et al., 2012). Importantly, these images can also be manipulated to induce stability of a particular percept, for example moving bistable stimuli can be stabilized by motion of background elements (Kramer and Yantis, 1997) and the Necker cube, which elicits viewpoint ambiguity, can be manipulated with color enhancement of particular sides so that one viewpoint is predominantly perceived (Wang et al., 2013). This enables the investigation of perceptual priors and their volitional control.

We have previously shown that alterations in perceptual priors by short-term changes in environmental statistics are linked to adjusted ratios of bottom-up to top-down signal propagation in neural hierarchies that exhibit a pronounced age effect, with older adults less likely to adjust their beliefs (Moran et al., 2014). In the current study, we build upon these findings to test whether advanced age is associated with greater control of what is perceived.

The aim of the study was to establish a perceptual preference based on external stimulus manipulations and to use dynamic causal modeling (DCM) to assess changes in effective connectivity that arise from bias training. Our training consisted of a modified Rubin vase as a non-ambiguous image used to induce bias within subjects. We intended to elicit this effect to observe a change in percept duration in the younger individuals behaviorally. For older adults, we hypothesized that they would resist biasing by the training stimulus (Moran et al., 2014) and more actively control perceptual states when viewing bistable images. We were interested specifically in whether there was an age-dependence in post-training constructive perception.

## Materials and Methods

### Participants

A total of 30 participants (16 females) partook in our fMRI experiment. The average age of the participants was 44.9, ranging from 18–76. Participants were divided into two groups: a young cohort with an average age of 23.9 (*n* = 14, 18–29 years, 7 females) and an older cohort with an average age of 63.7 (*n* = 16, 54–76 years, 9 females). All were screened for MRI contraindication and psychiatric or neurological disorders, had normal or corrected-to-normal visual acuity, and were fluent in English. Study protocols were approved by the Virginia Tech Institutional Review Board and written informed consent was obtained from each participant. Participants were compensated for their time.

### Experimental Protocol

**Figure 1:**
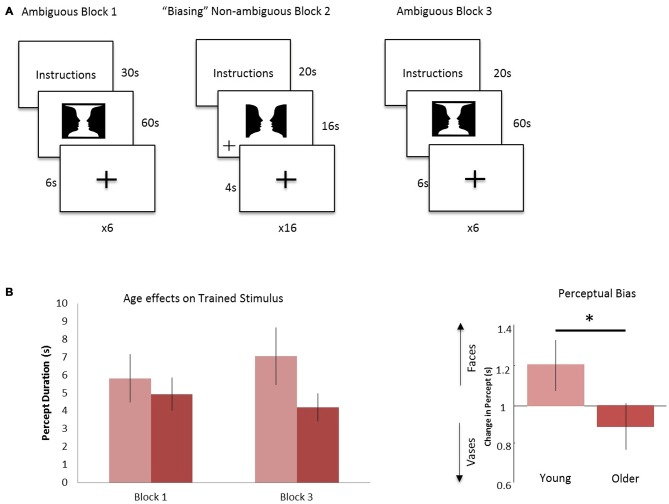
**Experimental design and age effects on trained stimulus. (A)** Block 1: the ambiguous Rubin vase was shown for 60 s, where participants indicated their perception, faces or vase, with a button press. This was repeated 6 times and each trial was separated by a 6 s fixation cross. Block 2: a non-ambiguous, modified Rubin vase was shown for 16 s, where participants indicated when the fixation-cross appeared on either the left or right of the image. This was repeated 16 times and each trial was separated by a 4 s fixation cross. Block 3 was identical in design to Block 1. **(B)** Left: the average duration in viewing faces (the biased percept) in Block 3 compared to Block 1 for the young cohort (light red) and older cohort (dark red). Right: the ratio of these durations—i.e., the perceptual biasing effect, was significantly different between the younger and older groups *p < 0.05.

Each participant received task instructions and completed an instruction quiz prior to the scanning session. The fMRI task consisted of three blocks: ambiguous Block 1, “Biasing” Non-ambiguous Block 2, and ambiguous Block 3 (Figure 1A). In ambiguous Block 1, the Rubin vase was presented for 60 s, followed by a fixation cross displayed for 6 s (Rubin, 1921). Participants were instructed to indicate via button press whether they perceived two faces or a vase initially as well as every time their perception switched over the 60-s trial. This experimental design was similar to that employed in Sterzer et al. (2009) in that participants were not given instructions to focus on one perception over the other. All button presses were recorded and this was repeated for a total of six trials. Participants were then shown a modified, non-ambiguous stimulus during the “Biasing” Non-ambiguous Block 2. This non-ambiguous stimulus was intended to explicitly portray two faces by modifying it in a way that the two faces was the most likely perception gained from looking at the stimulus. By presenting such an image, we intended to “train” or “bias” participants toward the perception of the faces vs. a vase when they viewed the ambiguous figure. The non-ambiguous stimulus was presented for a total of 16 s, followed by a fixation cross displayed for 4 s. This was repeated for a total of 16 trials. When the non-ambiguous stimuli were presented, a fixation cross would appear at random to either the left or right of the screen and participants were instructed to indicate via button press when the fixation cross appeared. In Ambiguous Block 3, participants were again presented the ambiguous Rubin vase image for 60 s, followed by a fixation cross displayed for 6 s and instructed to indicate via button press their initial perception and their subsequent perceptual switches. This repeated for a total of six trials. To summarize: in two blocks (Blocks 1 and 3), we showed participants a non-modified ambiguous Rubin vase figure. The non-ambiguous block (block 2) was the “training” block in which the participant was shown a modified version of the Rubin vase diagram eliciting a stable perception showing two faces, where the top and bottom borders were removed. This was a similar modification to the image as presented in Wang et al. (2013). The non-ambiguous image was also chosen as a result of pilot data (not reported) which suggested the Rubin image modified to elicit a face-bias was a stronger non-ambiguous image than the Rubin image modified to elicit a vase-bias.

Button presses indicating percept switches, their times, and perceptual durations were recorded for behavioral data analysis. Total percept duration throughout the trials and average percept duration for each perception, i.e., face or vase, was analyzed across age and block (pre- vs. post-training).

### fMRI Data Acquisition

Anatomical and functional images were acquired using a 3-T Siemens MAGNETOM Trio scanner. High-resolution T1-weighted structural images were collected using MPRAGE sequence with a repetition time (TR) = 1200 ms, echo time (TE) = 2.66 ms, field of view (FOV) = 245 mm, 1.0 mm slice thickness. Echo planar image data were acquired with a TR of 2000 ms, TE = 25 ms, field of view (FOV) = 220 mm, with 37 slices acquired at a slice thickness of 4.0 mm. Slices were oriented 30° superior-caudal to the plane through the anterior and posterior commissures to reduce signal drop-out. Headphones were used to reduce scanner noise. Participants used a mirror to view the stimuli projected behind them in the scanner. Participants were provided with additional items such as blankets and noise-cancelling ear plugs upon request.

### fMRI Data Analysis

Preprocessing and data analysis were performed using statistical parametric mapping software implemented in Matlab (SPM12b beta; Wellcome Trust Centre for Neuroimaging, London, UK). The first five functional images of the acquisition were discarded to allow for equilibrium magnetization. The mean scan was used as the reference for EPI blood-oxygen-level dependent (BOLD) images which were realigned with a six parameter spatial transformation. The structural image was co-registered to the mean resliced image. The unified segmentation routine was then used to perform segmentation bias correction and spatial normalization. Images were normalized to MNI space using the ICBM template. Then, the data was smoothed using a kernel with 8 mm full-width at half maximum (FWHM).

Individual participant BOLD responses were analyzed using a General Linear Model (GLM). There were nine total regressors: (1) ambiguous stimuli presentation Ambiguous Block 1; (2) ambiguous stimuli presentation Ambiguous Block 3; (3) non-ambiguous image presentation Non-ambiguous Block 2; (4) button press responses for Block 1; (5) pre-switch event, a 2000 ms time period immediately prior to button press, during Block 1; (6) button press responses for Block 3; (7) pre-switch event, a 2000 ms time period immediately prior to button press, during Block 3; (8) button press responses in Block 2; and (9) pre-press, 2000 ms prior to button press, during the Block 2. All regressors were convolved with a canonical hemodynamic response function. In the first level GLM, estimated motion parameters were used as nuisance regressors. Once all regressors for all individual GLMs had been created, contrasts were created at the first-level to identify activation differences between ambiguous and non-ambiguous stimuli and between the pre- and post-training ambiguous stimuli. We assigned 2000 ms prior to the button press as the “pre-switch” event. This was motivated by previous research suggesting that subjective decisions can be observed in fMRI activity up to 10 s prior to a motor report (Soon et al., 2008).

We then used a summary statistic approach to assess group-level whole-brain peak activations to identify regions of interest. An F-contrast was used to identify positive or negative responses to the ambiguous stimuli compared to non-ambiguous stimuli (Table 1). An F-contrast was also applied to identify training effects—examining positive or negative response differences to ambiguous stimuli before (Block 1) and after biasing (Block 3; Table 1). A 2 × 2 analysis of variance (ANOVA) was preformed to test interactions between age and training effects as well.

**Table 1:** **fMRI second level group statistics: effects of ambiguity and age correlations**.

Peak activation region (MNI)	*X*	*Y*	*Z*	*F* statistic	*P*_uncorrected_	*P*_FWE-corrected_
**(A) Significant voxels with positive or negative response to onset of ambiguous vs. non-ambiguous images, unmasked, extended threshold 10 voxels**
R Lingual	6	−68	−4	6.26	0	0
R Sup occipital	14	−96	18	5.99	0	0
L Cerebelum	−25	−58	−25	5.92	0	0
L Mid temporal	−52	−48	14	5.81	0	0
L Angular	−42	−64	36	5.47	0	0.001
**(B) Significant voxels with positive or negative response to onset of ambiguous images Block 1 vs. Block 3, unmasked, extended threshold 10 voxels**
L Lingual	−6	−68	−2	5.88	0	0
R Lingual	6	−64	−2	5.85	0	0
R Cuneus	14	−96	16	5.83	0	0
L Precentral	−60	2	28	5.83	0	0
L Mid temporal	−50	−60	−2	5.78	0	0
**(C) Significant voxels with positive or negative response to onset of ambiguous vs. non-ambiguous images, masked inclusively with a negative correlation contrast of age, extended threshold 10 voxels: Lingual gyrus and Precuneus**
Lingual	−6	−68	−2	5.90	0	0
L Precuneus	−12	−70	36	5.17	0	0.004
**Significant voxels with positive or negative response to onset of ambiguous vs. non-ambiguous images, masked inclusively with a positive correlation contrast of age, extended threshold 10 voxels: Mid Temporal gyrus and Inferior Orbitofrontal cortex**
R Mid temp	50	30	−6	4.94	0	0.011
R Inf orb	62	−22	−6	4.85	0	0.016

*(A) The peak activations were identified as positive or negative response to the onset of ambiguous compared to non-ambiguous images and image onsets of ambiguous images before and after biasing. Both comparisons were unmasked and extended thresholds were at 20 voxels. (B) The effects of perceptual biases—comparing post and pre-training responses to ambiguous stimuli. (C) The peak activations with positive or negative response to onset of ambiguous compared to non-ambiguous images were used to mask age covariation. Lingual and precuneus activations were found when testing for decreasing with age. Right middle temporal gyrus and right inferior orbitofrontal cortex activations were found with a covariate of increasing age*.

### Dynamic Causal Modeling

DCM for fMRI provides a model-based investigation of effective connectivity (Friston et al., 2003), where effective connectivity represents directional and modulatory interactions between multiple brain regions using separate neuronal and hemodynamic parameterizations. At the neuronal level the DCMs comprise a set of differential equations with parameters that control the drive of external inputs and of inter-regional neuronal influences. Given our interests in endogenous drivers of perceptual switches, we applied stochastic DCM for fMRIs which explicitly parameterizes non-stimulus linked fluctuations in neuronal activity (Li et al., 2011). We chose this over the alternative counterpart, deterministic DCM, due to its ability to parameterize and formally incorporate random neuronal fluctuations (Friston et al., 2014). Bayesian Model Selection was applied to find the best—most probable—model to explain the observed hemodynamics (Stephan et al., 2007). Our aim was to identify the neuronal connections associated with perceptual changes—i.e., pre-switch events.

We used our second-level summary statistics to identify regions of interest which responded differentially to ambiguous and non-ambiguous stimuli. We further used two age covariates to identify within these regions, specific nodes that exhibited positive and negative correlations with age. The regions of interest (ROIs) were identified around the group peak coordinates of the Lingual gyrus (LIN) [−6 −68 −2] and the Precuneus (PRE) [−12 −70 36]-these regions exhibited a negative correlation with age. ROIs were identified around the group peak coordinates of the Middle Temporal gyrus (MTG) [50 30 −6] and Inferior Orbitofrontal Cortex (IOF) [62 −22 −6]-with a positive correlation with age (group peaks are summarized in Table 1).

Given these coordinates, we extracted BOLD time series from each participant’s fMRI data individually. Time series were extracted using an F-contrast mask that tested for differences between ambiguous and non-ambiguous stimuli with a *p*-value threshold of *p* < 0.05, uncorrected with a sphere radius of 8 mm (note: *p*-values here are used to define the voxel cluster from which the principal eigenvariate will be extracted, they are not involved in the final DCM statistics). The principal eigenvariate within a sphere of 8 mm was extracted for the model-based analysis. To correct for confounding motion and button-press contributions to our ROI time series, these extractions were corrected for “effects of interest” using an F contrast to partition data variance in order to incorporate effects from just four regressors including: (1) “Ambiguous Block 1”, (2) “Ambiguous Block 3”, (5) “Pre-Switch Block 1” and (7) “Pre-Switch Block 3”. By using this F contrast, we are partitioning out any effects that could be due to all the other regressors, which include head motion and button presses.

To test the effective connections across the network we constructed four models of potential interactions among our four regions of interest. There were intrinsic connections within all regions and between all regions (DCM’s A matrix), except for the IOF and MTG. Inputs from ambiguous stimuli onsets drove all regions (DCM’s C matrix). Modulatory connections (DCM’s B matrix) were used to test network connections associated with pre-switch events. In model 1 we placed these modulations only on bottom-up connections for block 1 (LIN to MTG, LIN to IOF, PRE to MTG, and PRE to IOF) and only on top-down connections for post-training block 3 (MTG to LIN, MTG to PRE, IOF to LIN, IOF to PRE). For model 2 we allowed modulation of pre-switch events for pre and post training blocks on both sets of bottom-up and top-down connections. For model 3 we allowed modulation of pre-switch events only on bottom-up connections. Finally, for model 4 we allowed modulation of pre-switch events only on top-down connections.

## Results

### Behavioral Effects of Biasing

Behavioral data were analyzed to test for perceptual biasing effects. For this we compared the average duration of perception of trained stimulus (i.e., two faces, see Figure 1A) between pre- and post-training blocks. All percepts throughout the six 60-s trials were examined, regardless of number of switches made within the trial and of the initial percept. Furthermore, we ensured that all participants had at least three or more switches within a single trial of 60 s. From these data we established a simple bias ratio—the ratio of average time durations for perceptions where the stimulus was viewed as two faces for post-training (block 3) relative to pre-training (block 1; Figure 1). Although we were not interested in the initial percept at each trial during the ambiguous blocks, it is important to note that there was no significant difference on the effect of training or age in the initial percept.

Overall, our hypothesized effects of age on biasing were evidenced. Average percept duration of the trained stimuli was similar between the young and older group (Figure 1B), however, during the post-training block, the average duration of the “faces” percept significantly differed between the young and older cohort (young (*n* = 14): 7064 ms; older (*n* = 16): 4209 ms) showing a positive bias for the trained stimulus for the young relative to older cohort (*p* < 0.05, Figure 1B). There was a medium effect size in this comparison (Cohen’s *d* = 0.6). In addition, analysis of total percept duration for vases compared to faces post-training showed preference in older individuals towards the novel, non-trained percept significantly (*p* < 0.001). These data demonstrate a preference toward the novel or untrained percept in block 3 for the older cohort relative to a trained or biased prior in the younger cohort.

### SPM Analysis

**Figure 2:**
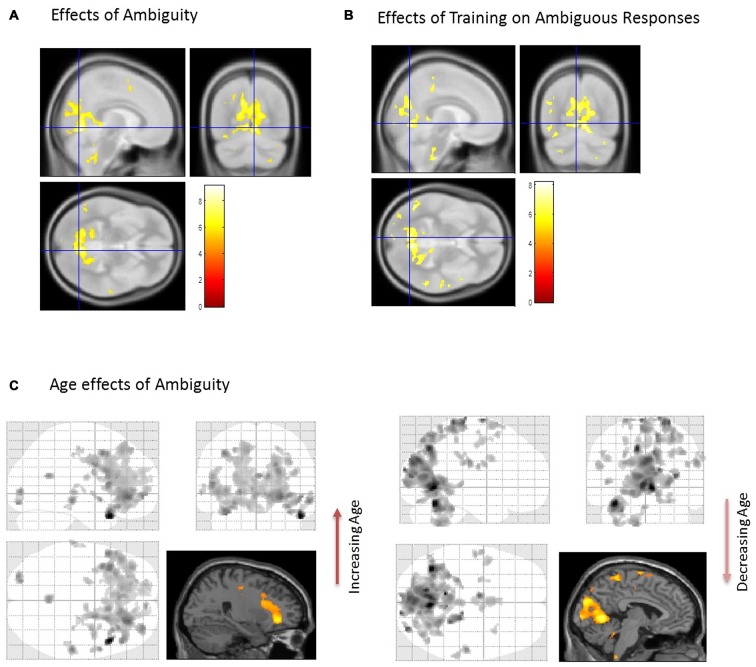
**Brain activations associated with the perception of ambiguous stimulus. (A)** When comparing Ambiguous and non-Ambiguous stimuli the overall effect was seen with a group peak activation in the R Lingual [6 −68 −4] (*p* < 0.05, family wise error (FWE) corrected). **(B)** Comparison of ambiguous stimuli before (Block 1) and after training (Block 3). When comparing the two ambiguous blocks (1 and 3) to measure the effect of the “biasing” block. Here, similar regions in parietal and visual cortices predominated with significant effects also observed in the right anterior temporal cortex (*p* < 0.05 *FWE* corrected, Table 1). **(C)** Using the regions differentially active to ambiguous vs. non-ambiguous stimuli as a mask **(A)** we then found activations that positively correlated with age in anterior regions (Right orbitofrontal and anterior temporal lobe, top image, *p* < 0.001, uncorrected, Table 1). In contrast regions negatively correlated with age were observed posteriorly (cluster peaks in lingual gyrus and precuneus, bottom image, *p* < 0.001, uncorrected, Table 1). For extracting our ROIs, application of F-contrasts as inclusive masks and these regions were present at *p* < 0.05, *FWE* corrected (image not shown).

First, we identified brain regions that exhibited significant effects of ambiguity; that is, we tested ambiguous relative to non-ambiguous blocks over the whole brain using an F-contrast. Comparing the responses to these stimuli we observed significant activation across a distributed brain network, with large activations in visual and parietal cortices—voxel peak in the LIN [6 −68 −4; *x*, *y*, *z* MNI coordinates] (*p* < 0.05, Family Wise Error (FWE) corrected, Table 1, Figure 2A). We then used this contrast as a mask to test for age dependencies within the regions that exhibited ambiguity effects and found negative correlations with ambiguity-related activations in visual and parietal cortices, with a peak in the LIN at [−6, −68, −2], (*p* < 0.05 FWE, Figure 2C). In contrast, positive correlations with age were found in frontal and temporal cortices with a peak in the anterior middle temporal gyrus [50, 30, −6] (*p* < 0.05, FWE, Table 1, Figure 2C).

We assessed group level interactions between training and age using a 2 × 2 ANOVA. An interaction of age and training was seen in posterior regions with peak activation in the right LIN [4 −64 6] (*p* < 0.05, FWE). To unpack this result we performed a “simple main effects” analysis specifically for training in Figure 2B. Here, we tested only for the effects of the non-ambiguous training block we compared ambiguous responses pre and post training. This contrast showed that similar regions exhibited the biasing effects as previous seen for ambiguous processing generally (ambiguous pre-trained vs. ambiguous post-training), including LIN, PRE and middle temporal cortices (*p* < 0.05, FWE, Table 1). Furthermore, we tested for other covariates that may be implicated in the context of psychopathology, including gender and education. These covariates did not show any significant effects in activation between male or female participants or in terms of education level categorized by some high school, high school graduate, some college, college graduate (data not shown).

### DCM of Ambiguous Visual Processing and Age-Related Connectivity Effects

**Figure 3:**
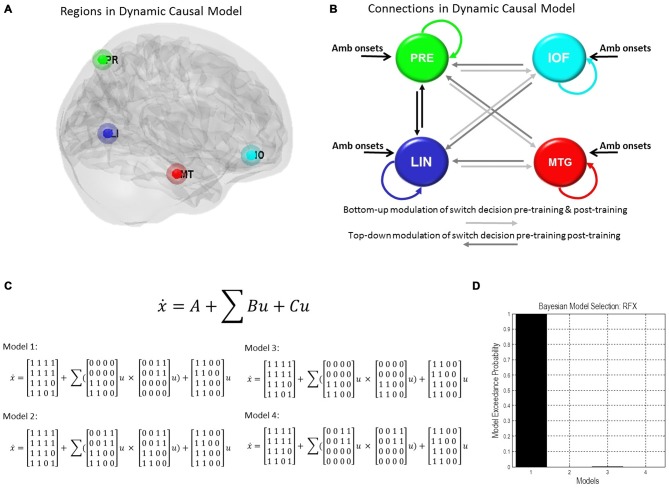
**Dynamic causal model and Bayesian model selection. (A)** Sources for the Dynamic Causal Modelings (DCMs) were obtained from the second level analysis, displayed here. Regions of interest were identified around the group peak coordinates for Lingual [−6 −68 −2], Precuneus [−12 −70 36], Mid Temporal [50 30 −6], and Inf Orbitofrontal [62 −22 −6]. **(B)** The four regions of interest (ROIs) were used to create a stochastic DCM. There were intrinsic connections within all regions and between all regions, except for the inferior orbitofrontal cortex (IOF) and middle temporal gyrus (MTG). **(B)** Inputs from ambiguous image onsets entered all regions. The modulations in connections associated with pre-switch events were tested using Bayesian model comparison. In model 1 pre-switch connections in the pre-training block were confined to bottom-up connections (light gray), i.e., from Lingual and PRE to Inferior Orbitofrontal and Mid Temporal Lobe. While in model 1 post-training switches were modeled via top-down connection modulations only (dark gray), i.e., from Inferior Orbitofrontal and Mid Temporal Lobe to Lingual and PRE. Model 2 comprised pre-switch, pre- and post-training modulations in both directions. Model 3 consisted of bottom-up pre-switch modulations for both pre- and post-training modulations. Finally, model 4 involved of top-down pre-switch modulations for both pre- and post-training modulations. **(C)** We also display the equations used to define each of these models. A is the intrinsic connection parameters matrix. B is the input-dependent or modulatory connection parameter matrix. z denotes the regions. C is the extrinsic influences or input connection parameter matrix. u Represents the inputs. **(D)** Bayesian model comparison revealed that both younger and older cohorts preferred model 1 (see “Results” Section) and these fixed effects were consistent across most subjects. Here, we illustrate the exceedance probabilities for a comparison including all models from both age groups.

We used those activations associated with ambiguous compared to non-ambiguous stimuli to study perceptual belief networks using DCM (see “Materials and Methods” Section, Figure 3A). We were particularly interested in the mechanisms subtending switches in subjective perceptual beliefs and the effects bias training had on the network. To analyze switch responses we defined “pre-switch events”, a 2000 ms period immediately prior to a button press indicating the percept had switched. We chose this timing due to the possibility of active networks present before the action of a button press, without overlapping button press responses. This was motivated by previous research suggesting that subjective decisions can be observed in fMRI activity up to 10 s prior to a motor report (Soon et al., 2008). Our network comprised four regions including LIN, PRE, mid-temporal gyrus (MTG) and IOF, with intrinsic connections arranged reciprocally among these regions (with the exception of IOF to MTG). The percent variances explained by our fMRI data in the four extracted principal eigenvariates over an 8 mm-radius sphere were determined and averages across all subjects were calculated (Table 2).

**Table 2:** **Average percent variation explained in regions of interest (ROIs)**.

	Average percent variance ± SEM
LIN	79.00% ± 1.91
PRE	79.18% ± 1.89
MTG	74.75% ± 2.06
IOF	77.58% ± 2.32

*For our DCMs, we extracted the principal eigenvariate for an 8 mm-radius sphere around a group peak at the four regions of interest (LIN: [−6, −68, −2]; PRE: [−12, −70, 36]; MTG: [50, 30, −6]; IOF: [62, −22, −6]) at *p* < 0.05, uncorrected. This principal eigenvariates explain the above percent variances on average over each region for all subjects. The table shows average ± standard error mean of these regions across all 30 subjects*.

We constructed four models to test for training-related differences in top-down vs. bottom-up perceptual control. In model 1, pre-switch modulations during the pre-training block were confined to bottom-up connections and pre-switch modulations during the post-training block were confined to top-down connections. In model 2, we allowed for both bottom-up and top-down pre-switch modulations in both pre- and post-training blocks (Figure 3B). In model 3, only bottom-up pre-switch modulations were present in both pre- and post-training blocks. In contrast, in model 4, only top-down pre-switch modulations were present in both pre- and post-training blocks. We show the equations representing the models (Figure 3C). Using a random-effects Bayesian model comparison across participants and within each cohort separately we found that both the young and older cohorts preferred model 1 (with a model exceedance probability (MEP) of 0.9993 for all subjects, Figure 3D, MEP = 0.9882 in the young cohort, and MEP = 0.9494 in the older cohort). The effect across individuals was consistent with 11 participants in the younger cohort preferring model 1, and the other three preferring model 3. Nine participants in the older cohort preferred model 1, five preferred model 4, and two preferred model 3.

**Figure 4:**
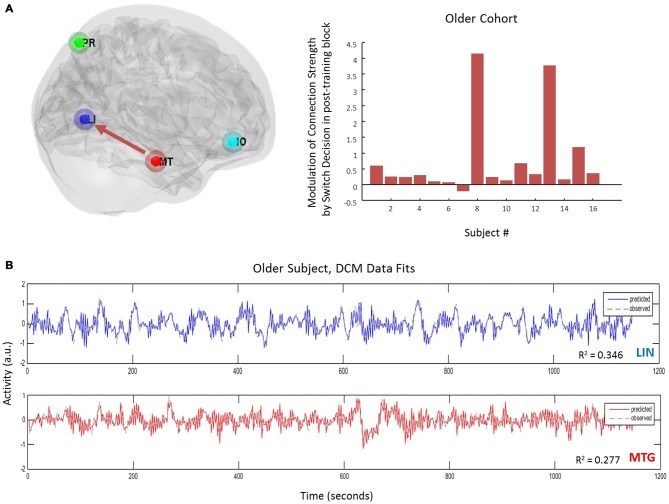
**Age-effects on connectivity mediating volitional perception. (A)** Only in the older cohort did we see pre-switch modulations of effective connectivity. Specifically we observed an emergence of a top-down control connection (MTG → LIN) following the bias or training block. **(B)** DCM fits here from one subject and displayed for two regions accurately recapitulate the extracted time-series.

Equipped with this winning model we tested for training or biasing effects within each cohort. To test for effect size, the average coefficients of determination for the model fit were determined in each cohort. There is a medium effect size for the lingual region in the young cohort, a medium effect size for the middle temporal regions in both cohorts, and a small effect size for PRE and inferior orbitofrontal regions (Table 3). Interestingly, we observed that the young cohort exhibited no significant modulations in connections related to training. Rather, we found that the arrangement of driving inputs differed between pre- and post-training. Specifically, the initial pre-training block was associated with a negative driving input to both lingual (student’s *t* test; *p* = 0.0016) and PRE sources (*p* = 0.014), while in the post-training block these negative driving inputs were confined to the lingual source only (*p* = 0.0017). These negative driving inputs will suppress endogenous noise in each region under the stochastic DCM. In the older cohort however significant effects of training were observed—with the emergence of a significant top-down connection from the middle temporal gyrus to LIN on the post-training block (*p* < 0.05; Figure 4A). This cohort also exhibited negative input drive into lingual and frontal sources post training (*p* < 0.05). Within both cohorts, the DCMs adequately recapitulated the measured data features (Figure 4B).

**Table 3:** **Average coefficient of determination for DCM fits in ROIs**.

	Average coefficient of determination (R^2^) ± SEM			
	LIN	PRE	MTG	IOF
Young (*n* = 14)	0.30 ± 0.03	0.12 ± 0.02	0.29 ± 0.04	0.076 ± 0.02
Older (*n* = 16)	0.20 ± 0.02	0.11 ± 0.03	0.28 ± 0.03	0.073 ± 0.02
Overall	0.24 ± 0.02	0.12 ± 0.02	0.29 ± 0.02	0.074 ± 0.01

*Coefficients of determination for DCM fits were calculated for all 30 subjects for the winning model. We present these as effect size for our DCM data extraction and model fit. We compared predicted response with observed response. Coefficients of determination show a medium effect size for the LIN region in the young cohort, a medium effect size for the MTG region in both cohorts, and a small effect size for PRE and IOF regions*.

Hemodynamic changes with age may alter BOLD activity and contribute to second-level group statistics (Tsvetanov et al., 2015). With DCM we were able to separate the hemodynamic parameters and test whether they exhibited age-dependent effects. However no effects of age on hemodynamic parameters were observed where we tested decay and transit time differences between the two age groups for all four regions (*p* > 0.1 uncorrected for eight tests).

## Discussion

Despite theoretical and imaging-driven advances in understanding bistable perception, its interaction with an aging neurobiology has received little attention. Motivated by the ubiquitous role age plays in psychopathological status (Häfner et al., 1998; Ho et al., 2000; Jeste et al., 2003; Topor et al., 2013; Lin et al., 2006), the present study addresses the age-dependency of neuronal connectivity underlying volitional control of perceptual beliefs. In our study, we investigated the brain regions associated with fluctuating perceptual content, whether these brain regions interact during perceptual rivalry, and how stimulus-driven biasing can affect subsequent subjective perceptual beliefs and neuronal connectivity. In our study, we used the Rubin vase diagram and manipulated the image in order to bias perception and tested the underlying processing networks using fMRI and DCM. In summary our findings reveal that consistent with our hypothesized training effect, older cohorts exhibited a resistance to perceptual biasing compared to the younger cohort and these effects were found to be mediated by an increase in top-down connections from temporal to visual cortical sources post training.

Our study was motivated by predictive coding theories of cortico-cortical interactions which has been explored recently in the context of visual illusory processing (Brown and Friston, 2012; Chopin and Mamassian, 2012). Our aim was to determine whether prior beliefs could resist external manipulation in an age-dependent manner. Our paradigm was suited to this connectivity hypothesis given recent work by Kok et al. (2016) who show that top-down connections selectively activate early visual regions during the perception of illusory figures such as the Kanizsa stimulus. In our study, we used the non-ambiguous block for training to test whether inference networks within the brain became more robust to environmental perturbations as we age. This fits within larger theoretical frameworks such as the Free-energy principle (Brown and Friston, 2012), which appeals to the Bayesian brain hypothesis and laminar specific connectivity which optimizes to better predict future sensory inputs (Moran et al., 2014). With this in mind, we suggest that perceptual switches in the aging population can be described as changes in connectivity between regions, generated by an internal predictive model. In the context of visual processing and perceptual competition, binocular rivalry is another phenomenon explained in the framework of a brain that is engaged in Bayesian inference (Hohwy et al., 2008). Furthermore our motivation for this framework relates to psychopathology where studies such as Shergill et al. (2005) have implicated predictive coding abnormalities in diseases such as schizophrenia.

Whole-brain analysis from the fMRI study identified a network of cortical regions involved in viewing the ambiguous figures that included the LIN and precuneus, regions typically associated with perceptual changes in ambiguous figures (Sterzer and Kleinschmidt, 2007; Wang et al., 2013). Within these activated regions we found a striking correlation with aging, as age increases the ambiguity-associated activations predominated in anterior regions, while younger age was associated with greater posterior activity. This is consistent with general aging effects observed in fMRI-neurocognitive experiments which demonstrate a posterior to anterior shift in activation (PASA) patterns (Cabeza, 2001; Davis et al., 2008). With regards to PASA, there is reduced neural specialization in the visual cortex with age as well as an increase in distributed processing in frontal areas (Cabeza, 2001), with these anterior shifts noted in visual processing tasks (Ansado et al., 2012). However, such a paradigm has not been considered in bistable perception visual processing, making our study unique in that matter. In our study, we show that this shift to anterior regions of the brain can be associated with visual processing and perceptual control and not attributed to any specific default network, which has been shown to undergo reallocation with aging as a compensatory mechanism (Davis et al., 2008). We are unable to provide evidence for or against a compensatory mechanism in our study since we do not have a metric of “good” or “poor” performance. Instead, we are interested in Bayesian predictive coding leading to differences in connectivity. Exploring our activations using DCM we found that younger participants did recruit frontal regions during ambiguous stimulus processing but that this dropped offline following a biasing session. In contrast, our older cohorts resisted biasing and furthermore recruited top-down connections to control their perceptual beliefs following training. In the context of psychopathology it may be useful to control perceptual beliefs internally and to resist model updating based on spurious environmental stimuli. An inaccurate assignment of one’s environmental experiences may contribute to the underlying pathology in diseases such as schizophrenia (Kapur, 2003).

Previous behavioral studies using binocular rivalry have shown that perceptual stability increases with increasing age (Ukai et al., 2003; Beers et al., 2013). However, binocular rivalry, compared to bistable perception with ambiguous figures, involves a more automatic and stimulus driven form of visual competition occurring at the lower levels of the visual pathway (Tong et al., 2006). We do not assume that our findings extend to studies of binocular rivalry. Bistable perception with ambiguous figures occurs at a higher level in the visual pathway (Tong and Engel, 2001). This provides a method of intentional control, making it more suitable for the larger goal of our analysis, which is the study of the active process of perception. Using multisensory sound flash-illusions, studies have also demonstrated that aging presents with stronger illusory percepts compared to younger adults (DeLoss et al., 2013), but that training to avoid the temporal overlap illusion can be accomplished by older cohorts (Setti et al., 2014). Few studies however have sought to establish the neural correlates of these effects. In our study we used stochastic DCM for fMRI (Daunizeau et al., 2011; Li et al., 2011) in order to account for the internally-generated dynamics that cause endogenous percept fluctuations as well as task-dependent changes (deterministic effects; Friston et al., 2014). This is in contradistinction to other spectral DCMs which may present a more accurate and parsimonious account of connectivity in studies examining complete resting or stationary states (Razi et al., 2015). The optimized parameter sets of our stochastic models revealed interesting dynamics particularly in the driving inputs (Friston et al., 2003). We found that negative driving inputs were observed in posterior and frontal sources for the older subjects post-training whereas for the younger subjects these patterns were seen pre-training with a dropout of frontal inhibitory drive post-training. The polarity of these driving inputs are reasonable in the setting of stochastic DCMs since they would dampen endogenous noisy fluctuations in their respective regions and in the case of the older cohort enable top-down control via long-range connections.

Our results complement previous studies involving bistable perception, which have shown a decline in attentional selection of low-salient stimuli (Tsvetanov et al., 2013). Additionally, Aydin et al. (2013) examined perceptual switching of the Rubin vase showing that older individuals are less likely to attend to visual stimuli after holding a specific percept. In fact, the older group prefers the novel percept. However, we do not use distractors or perceptual holding in our experiment but rather assess control in the context of biasing effects.

Overall, our analysis provides a holistic account of bistable perceptual processing in aging given the combination of fMRI and stochastic DCMs. Our observed network involving the frontal and temporal regions was derived from our whole brain analysis. Our regions in these models are supported by previous research suggesting significant modulation of inferior frontal cortex to medial temporal regions during the perceptual transitions of the ambiguous rotating Lissajous figure (Weilnhammer et al., 2013). All of our four ROIs have been shown to respond to bistable percepts in previous studies (Wang et al., 2013). An electroencephalogram (EEG) study on bistable perception using ambiguous images showed activity in the posterior visual regions in addition to higher-order fronto-parietal and temporal regions of the brain (Britz et al., 2009). In terms of psychopathology, frontal and temporal cortices, specifically the inferior frontal gyrus and superior temporal gyrus, is implicated in schizophrenia showing altered connectivity in resting state fMRI study (Zaytseva et al., 2015). Orbitofrontal cortices and middle temporal gyrus are furthermore areas of disruption in perspective-taking tasks in schizophrenia (Eack et al., 2013). Brain networks such as the default network or salience network may play a role in bistable perception and show differences in age. Future work can use task-based independent component analysis (ICA; Hyett et al., 2015; Tsvetanov et al., 2015) to characterize the network topology in control to better understand perceptual changes.

Limitations of the study include other covariates that are affected with normal aging. For example, potential time differences may exist in the pre-switch event with age. In our study, we allotted the same pre-switch period duration for both the younger and older group. Given that our analysis relies on subjective recording of perceptual switches, this is an inherent limitation in the study of bistable perception since the only objective marker to assume a change in perception is the button press. Other limitations of the study include putative effects on bistability not accounted for in our design including eye position (Einhäuser et al., 2004) or attention (van Ee et al., 2005). Future studies could address these and more fine-grained features of aging control dynamics, using electrophysiological DCMs (Legon et al., 2015). For example, GABA levels in the visual cortex have been linked to bistable perception, with higher concentrations resulting in slower perceptual dynamics (van Loon et al., 2013)-an effect used to simulate aging differences in computational modeling studies of multistable perception (Hoshino, 2013). These simple visual paradigms may uncover further neurobiological correlates of perceptual control, and provide important clues for developmental and aging dependencies in psychopathology.

## Author Contributions

ED and RJM designed the experiment, performed analysis. ED, SEA and ABS collected the data. ED, SEA, ABS and RJM prepared the manuscript.

## Funding

This work was supported by a start-up grant from VTCRI to RJM.

## Conflict of Interest Statement

The authors declare that the research was conducted in the absence of any commercial or financial relationships that could be construed as a potential conflict of interest.

## References

[B1] Ansado J. Monchi O. Ennabil N. Faure S. Joanette Y. (2012). Load-dependent posterior-anterior shift in aging in complex visual selective attention situations. Brain Res. 1454, 14–22. 10.1016/j.brainres.2012.02.061 22483790

[B2] Aydin S. Strang N. C. Manahilov V. (2013). Age-related deficits in attentional control of perceptual rivalry. Vision Res. 77, 32–40. 10.1016/j.visres.2012.11.010 23206550

[B3] Beers A. M. Bennett P. J. Sekuler A. B. (2013). Age-related effects of size and contrast on binocular rivalry. J. Vis. 13, 546–546. 10.1167/13.9.546

[B4] Blake R. (1989). A neural theory of binocular rivalry. Psychol. Rev. 96, 145–167. 10.1037/0033-295x.96.1.145 2648445

[B54] Britz J. Landis T. Michel C. M. (2009). Right parietal brain activity precedes perceptual alternation of bistable stimuli. Cereb. Cortex 19, 55–65. 10.1093/cercor/bhn056 18424780

[B5] Brown H. Friston K. J. (2012). Free-energy and illusions: the cornsweet effect. Front. Psychol. 3:43. 10.3389/fpsyg.2012.00043 22393327 PMC3289982

[B6] Cabeza R. (2001). Cognitive neuroscience of aging: contributions of functional neuroimaging. Scand. J. Psychol. 42, 277–286. 10.1111/1467-9450.00237 11501741

[B7] Cardin V. Friston K. J. Zeki S. (2011). Top-down modulations in the visual form pathway revealed with dynamic causal modeling. Cereb. Cortex 21, 550–562. 10.1093/cercor/bhq122 20621984 PMC3041008

[B8] Carlson G. A. Bromet E. J. Driessens C. Mojtabai R. Schwartz J. E. (2002). Age at onset, childhood psychopathology and 2-year outcome in psychotic bipolar disorder. Am. J. Psychiatry 159, 307–309. 10.1176/appi.ajp.159.2.307 11823277

[B9] Carter T. D. C. Mundo E. Parikh S. V. Kennedy J. L. (2003). Early age at onset as a risk factor for poor outcome of bipolar disorder. J. Psychiatr. Res. 37, 297–303. 10.1016/s0022-3956(03)00052-9 12765852

[B10] Chopin A. Mamassian P. (2012). Predictive properties of visual adaptation. Curr. Biol. 22, 622–626. 10.1016/j.cub.2012.02.021 22386314

[B11] Daunizeau J. David O. Stephan K. E. (2011). Dynamic causal modelling: a critical review of the biophysical and statistical foundations. Neuroimage 58, 312–322. 10.1016/j.neuroimage.2009.11.062 19961941

[B12] Davis S. W. Dennis N. A. Daselaar S. M. Fleck M. S. Cabeza R. (2008). Qué PASA? the posterior-anterior shift in aging. Cereb. Cortex 18, 1201–1209. 10.1093/cercor/bhm155 17925295 PMC2760260

[B13] Dayan P. (1998). A hierarchical model of binocular rivalry. Neural Comput. 10, 1119–1135. 10.1162/089976698300017377 9654769

[B55] de Jong M. C. Brascamp J. W. Kemner C. van Ee R. Verstraten F. A. (2014). Implicit perceptual memory modulates early visual processing of ambiguous images. J. Neurosci. 34, 9970–9981. 10.1523/JNEUROSCI.2413-13.2014 25057199 PMC6608307

[B14] de Jong M. C. Knapen T. van Ee R. (2012). Opposite influence of perceptual memory on initial and prolonged perception of sensory ambiguity. PLoS One 7:e30595. 10.1371/journal.pone.0030595 22295095 PMC3266287

[B15] DeLoss D. J. Pierce R. S. Andersen G. J. (2013). Multisensory integration, aging and the sound-induced flash illusion. Psychol. Aging 28, 802–812. 10.1037/a0033289 23978009 PMC3778128

[B16] Dima D. Roiser J. P. Dietrich D. E. Bonnemann C. Lanfermann H. Emrich H. M.. (2009). Understanding why patients with schizophrenia do not perceive the hollow-mask illusion using dynamic causal modelling. Neuroimage 46, 1180–1186. 10.1016/j.neuroimage.2009.03.033 19327402

[B66] Dima D. Dietrich D. E. Dillo W. Emrich H. M. (2010). Impaired topdown processes in schizophrenia: a DCM study of ERPs. Neuroimage 52, 824–832. 10.1016/j.neuroimage.2009.12.086 20056155

[B56] Eack S. M. Wojtalik J. A. Newhill C. E. Keshavan M. S. Phillips M. L. (2013). Prefrontal cortical dysfunction during visual perspective-taking in schizophrenia. Schizophr. Res. 150, 491–497. 10.1016/j.schres.2013.08.022 24055199 PMC3825745

[B57] Einhäuser W. Martin K. A. König P. (2004). Are switches in perception of the Necker cube related to eye position? Eur. J. Neurosci. 20, 2811–2818. 10.1111/j.1460-9568.2004.03722.x 15548224

[B17] Foxe J. J. Murray M. M. Javitt D. C. (2005). Filling-in in schizophrenia: a high-density electrical mapping and source-analysis investigation of illusory contour processing. Cereb. Cortex 15, 1914–1927. 10.1093/cercor/bhi069 15772373

[B18] Friston K. J. Harrison L. Penny W. (2003). Dynamic causal modelling. Neuroimage 19, 1273–1302. 10.1016/s1053-8119(03)00202-7 12948688

[B19] Friston K. J. Kahan J. Biswal B. Razi A. (2014). A DCM for resting state fMRI. Neuroimage 94, 396–407. 10.1016/j.neuroimage.2013.12.009 24345387 PMC4073651

[B20] Gur R. E. Petty R. G. Turetsky B. I. Gur R. C. (1996). Schizophrenia throughout life: sex differences in severity and profile of symptoms. Schizophr. Res. 21, 1–12. 10.1016/0920-9964(96)00023-0 8864248

[B21] Häfner H. Hambrecht M. Löffler W. Munk-Jørgensen P. Riecher-Rössler A. (1998). Is schizophrenia a disorder of all ages? A comparison of first episodes and early course across the life-cycle. Psychol. Med. 28, 351–365. 10.1017/s0033291797006399 9572092

[B22] Ho B. C. Andreasen N. C. Flaum M. Nopoulos P. Miller D. (2000). Untreated initial psychosis: its relation to quality of life and symptom remission in first-episode schizophrenia. Am. J. Psychiatry 157, 808–815. 10.1176/appi.ajp.157.5.808 10784476

[B23] Hohwy J. Roepstorff A. Friston K. (2008). Predictive coding explains binocular rivalry: an epistemological review. Cognition 108, 687–701. 10.1016/j.cognition.2008.05.010 18649876

[B58] Hoshino O. (2013). Ambient GABA responsible for age-related changes in multistable perception. Neural Comput. 25, 1164–1190. 10.1162/NECO_a_00431 23470123

[B59] Hyett M. P. Breakspear M. J. Friston K. J. Guo C. C. Parker G. B. (2015). Disrupted effective connectivity of cortical systems supporting attention and interoception in melancholia. JAMA Psychiatry 72, 350–358. 10.1001/jamapsychiatry.2014.2490 25692565

[B24] Jeste D. V. Twamley E. W. Eyler Zorrilla L. T. Golshan S. Patterson T. L. Palmer B. W. (2003). Aging and outcome in schizophrenia. Acta Psychiatr. Scand. 107, 336–343. 10.1034/j.1600-0447.2003.01434.x 12752029

[B25] Kapur S. (2003). Psychosis as a state of aberrant salience: a framework linking biology, phenomenology and pharmacology in schizophrenia. Am. J. Psychiatry 160, 13–23. 10.1176/appi.ajp.160.1.13 12505794

[B26] Kersten D. Schrater P. R. (2002). “Pattern inference theory: a probabilistic approach to vision,” in Perception and the Physical World, eds Mausfeld R. Heyer D. (Chichester: John Wiley & Sons), 191–228.

[B27] Kloosterman N. A. Meindertsma T. Hillebrand A. van Dijk B. W. Lamme V. A. Donner T. H. (2015). Top-down modulation in human visual cortex predicts the stability of a perceptual illusion. J. Neurophysiol. 113, 1063–1076. 10.1152/jn.00338.2014 25411458 PMC4329440

[B28] Kok P. Bains L. J. van Mourik T. Norris D. G. de Lange F. P. (2016). Selective activation of the deep layers of the human primary visual cortex by top-down feedback. Curr. Biol. 26, 371–376. 10.1016/j.cub.2015.12.038 26832438

[B29] Kramer P. Yantis S. (1997). Perceptual grouping in space and time: evidence from the ternus display. Percept. Psychophys. 59, 87–99. 10.3758/bf03206851 9038411

[B60] Legon W. Punzell S. Dowlati E. Adams S. E. Stiles A. B. Moran R. J. (2015). Altered prefrontal excitation/inhibition balance and prefrontal output: markers of aging in human memory networks. Cereb. Cortex. pii:bhv200. 10.1093/cercor/bhv200 [Epub ahead of print]. 26400915

[B30] Leopold D. A. Logothetis N. K. (1999). Multistable phenomena: changing views in perception. Trends Cogn. Sci. 3, 254–264. 10.1016/s1364-6613(99)01332-7 10377540

[B31] Leopold D. A. Wilke M. Maier A. Logothetis N. K. (2002). Stable perception of visually ambiguous patterns. Nat. Neurosci. 5, 605–609. 10.1038/nn851 11992115

[B32] Li B. Daunizeau J. Stephan K. E. Penny W. Hu D. Friston K. (2011). Generalised filtering and stochastic DCM for fMRI. Neuroimage 58, 442–457. 10.1016/j.neuroimage.2011.01.085 21310247

[B33] Lin P. I. McInnis M. G. Potash J. B. Willour V. MacKinnon D. F. DePaulo J. R.. (2006). Clinical correlates and familial aggregation of age at onset in bipolar disorder. Am. J. Psychiatry 163, 240–246. 10.1176/appi.ajp.163.2.240 16449477

[B34] Malla A. Norman R. Schmitz N. Manchanda R. BÉChard-Evans L. Takhar J.. (2006). Predictors of rate and time to remission in first-episode psychosis: a two-year outcome study. Psychol. Med. 36, 649–658. 10.1017/s0033291706007379 16515734

[B35] Moran R. J. Symmonds M. Dolan R. J. Friston K. J. (2014). The brain ages optimally to model its environment: evidence from sensory learning over the adult lifespan. PLoS Comput. Biol. 10:e1003422. 10.1371/journal.pcbi.1003422 24465195 PMC3900375

[B36] Notredame C. E. Pins D. Deneve S. Jardri R. (2014). What visual illusions teach us about schizophrenia. Front. Integr. Neurosci. 8:63. 10.3389/fnint.2014.00063 25161614 PMC4130106

[B61] Razi A. Kahan J. Rees G. Friston K. J. (2015). Construct validation of a DCM for resting state fMRI. Neuroimage 106, 1–14. 10.1016/j.neuroimage.2014.11.027 25463471 PMC4295921

[B37] Rubin E. (1921). Visuell Wahrgenommene Figuren: Studien in Psychologischer Analyse. Copenhagen: Gyldendalske boghandel.

[B38] Schultz S. K. Miller D. D. Oliver S. E. Arndt S. Flaum M. Andreasen N. C. (1997). The life course of schizophrenia: age and symptom dimensions. Schizophr. Res. 23, 15–23. 10.1016/s0920-9964(96)00087-4 9050124

[B39] Setti A. Stapleton J. Leahy D. Walsh C. Kenny R. A. Newell F. N. (2014). Improving the efficiency of multisensory integration in older adults: audio-visual temporal discrimination training reduces susceptibility to the sound-induced flash illusion. Neuropsychologia 61, 259–268. 10.1016/j.neuropsychologia.2014.06.027 24983146

[B40] Shapiro A. Moreno-Bote R. Rubin N. Rinzel J. (2009). Balance between noise and adaptation in competition models of perceptual bistability. J. Comput. Neurosci. 27, 37–54. 10.1007/s10827-008-0125-3 19125318 PMC2913428

[B41] Shergill S. S. Samson G. Bays P. M. Frith C. D. Wolpert D. M. (2005). Evidence for sensory prediction deficits in schizophrenia. Am. J. Psychiatry 162, 2384–2386. 10.1176/appi.ajp.162.12.2384 16330607

[B42] Soon C. S. Brass M. Heinze H. J. Haynes J. D. (2008). Unconscious determinants of free decisions in the human brain. Nat. Neurosci. 11, 543–545. 10.1038/nn.2112 18408715

[B43] Stephan K. E. Weiskopf N. Drysdale P. M. Robinson P. A. Friston K. J. (2007). Comparing hemodynamic models with DCM. Neuroimage 38, 387–401. 10.1016/j.neuroimage.2007.07.040 17884583 PMC2636182

[B62] Sterzer P. Kleinschmidt A. (2007). A neural basis for inference in perceptual ambiguity. Proc. Natl. Acad. Sci. U S A 104, 323–328. 10.1073/pnas.0609006104 17190824 PMC1765459

[B44] Sterzer P. Kleinschmidt A. Rees G. (2009). The neural bases of multistable perception. Trends Cogn. Sci. 13, 310–318. 10.1016/j.tics.2009.04.006 19540794

[B45] Sundareswara R. Schrater P. R. (2008). Perceptual multistability predicted by search model for Bayesian decisions. J. Vis. 8, 12.1–12.19. 10.1167/8.5.12 18842083

[B46] Tong F. Engel S. A. (2001). Interocular rivalry revealed in the human cortical blind-spot representation. Nature 411, 195–199. 10.1038/35075583 11346796

[B47] Tong F. Meng M. Blake R. (2006). Neural bases of binocular rivalry. Trends Cogn. Sci. 10, 502–511. 10.1016/j.tics.2006.09.003 16997612

[B48] Topor D. R. Swenson L. Hunt J. I. Birmaher B. Strober M. Yen S.. (2013). Manic symptoms in youth with bipolar disorder: factor analysis by age of symptom onset and current age. J. Affect. Disord. 145, 409–412. 10.1016/j.jad.2012.06.024 23021377 PMC3535567

[B50] Tsvetanov K. A. Henson R. N. A. Tyler L. K. Davis S. W. Shafto M. A. Taylor J. R.. (2015). The effect of ageing on fMRI: correction for the confounding effects of vascular reactivity evaluated by joint fMRI and MEG in 335 adults. Hum. Brain Mapp. 36, 2248–2269. 10.1002/hbm.22768 25727740 PMC4730557

[B49] Tsvetanov K. A. Mevorach C. Allen H. Humphreys G. W. (2013). Age-related differences in selection by visual saliency. Atten. Percept. Psychophys 75, 1382–1394. 10.3758/s13414-013-0499-9 23812959

[B51] Ukai K. Ando H. Kuze J. (2003). Binocular rivalry alternation rate declines with age. Percept. Mot. Skills 97, 393–397. 10.2466/pms.97.5.393-397 14620224

[B52] van Ee R. van Dam L. C. J. Brouwer G. J. (2005). Voluntary control and the dynamics of perceptual bi-stability. Vision Res. 45, 41–55. 10.1016/j.visres.2004.07.030 15571737

[B63] van Loon A. M. Knapen T. Scholte H. S. St. John-Saaltink E. Donner T. H. Lamme V. A. (2013). GABA shapes the dynamics of bistable perception. Curr. Biol. 23, 823–827. 10.1016/j.cub.2013.03.067 23602476

[B53] Wang M. Arteaga D. He B. (2013). Brain mechanisms for simple perception and bistable perception. Proc. Natl. Acad. Sci. U S A 10, E3350–E3359. 10.1073/pnas.1221945110 23942129 PMC3761598

[B64] Weilnhammer V. A. Ludwig K. Hesselmann G. Sterzer P. (2013). Frontoparietal cortex mediates perceptual transitions in bistable perception. J. Neurosci. 33, 16009–16015. 10.1523/JNEUROSCI.1418-13.2013 24089505 PMC6618467

[B65] Zaytseva Y. Chan R. C. Pöppel E. Heinz A. (2015). Luria revisited: cognitive research in schizophrenia, past implications and future challenges. Philos. Ethics Humanit. Med. 10:4. 10.1186/s13010-015-0026-9 25886206 PMC4351688

